# EFL Teachers' Apprehension and L2 Students' Classroom Engagement

**DOI:** 10.3389/fpsyg.2021.758629

**Published:** 2021-09-29

**Authors:** Xiaodong Li

**Affiliations:** School of Foreign Studies, University of Science and Technology Beijing, Beijing, China

**Keywords:** apprehension, classroom engagement, English as a Foreign language, communication apprehension, class activities

## Abstract

Teachers' apprehension is an indispensable part of the educational context since it impacts the amount to which students are engaged in class activities. Although several studies have been carried out considering the role of students' stress in their engagement, it seems extremely vital to conduct such studies among teachers to measure the link between these two variables. In this study, the author has made endeavors to define one of the apprehension's categories named communication apprehension and the antecedents of teachers' apprehension. Then classroom engagement is discussed. Following that, the relevance between the two variables of this research is discussed. Finally, both implications and suggestions for further studies are dealt with.

## Introduction

Since teachers play a vital role in both students' classroom engagement and academic achievements, studies with such topics are of great importance. Prior studies have mostly highlighted the impact of Positive Psychology on students' engagement (Dewaele et al., 2019; MacIntyre et al., 2019; Wang et al., 2021) or the role of students' stress on their engagement (Gallagher, 2013). The aim of this study is to review the relationship between teachers' apprehension and students' classroom management. To this end, first of all, communication apprehension has been defined along with the reasons for teachers' apprehension. Then classroom engagement as well as its relationship with teachers' apprehension has been discussed, and finally further recommendations for future studies have been put forward.

## Background

### Teachers' Apprehension

#### The Definition of Apprehension and Its Types

Apprehension is conceptualized as the context-specific anxiety about the future, especially about handling something nasty or tough (Kyriacou, 2001). One of the features of apprehension is anticipating unpleasant or negative events. Another distinctive attribute of apprehension is that it is highly linked with fear or anxiety about communication.

Stress has always been viewed as a negative experience by which both teachers' self-esteem and well-being are threatened and it also impedes the learning process (Kyriacou and Sutcliffe, 1978). There are many stressors that are experienced in the educational arena, for instance, internal stressors. When a teacher is not good at communicative skills, when a teacher feels alone without being supported by his colleagues, and when a teacher is observed by the supervisors, the internal stressor is augmented. External stressors are triggered when a teacher is worried about establishing a good rapport with the students, when a teacher is not up-to-date with the latest teaching materials and also teaching tools, when a teacher has to deal with the lack of time for covering the subject-matter, taking a rest in break times, when a teacher has a hectic schedule and too much work to be done, and when he has to struggle with the students who are not motivated enough or they were absent (Kyriacou, 2001). One of the apprehension types that affects language learning is communication apprehension which is conceptualized as a type of shyness considered as fear of or anxiety about communication with people. Having trouble speaking in groups (oral communication anxiety) or in listening to or learning a spoken message are all indicators of communication apprehension. As it was proposed, communication apprehension has a detrimental impact on the process of language learning. Because language anxiety is one of the radical factors that controls the level of comprehensible input which is received by students, it has undeniably developed into a noticeable factor in determining how successful second language learning is (Horwitz et al., 1986). A scale to estimate teachers' apprehension (STAS) including 33 items, categorized into four groups: attitudinal factors, organizational factors, L2-related factors, and classroom management factors, were designed by Ghanizadeh et al. (2020) that revealed that the amount of apprehension experienced by a teacher is strongly correlated with teachers' identity and self-esteem they build for themselves. Some of the items of the above-mentioned scale are as follows: teachers feel worried when they feel pressurized before attending the class, teachers are afraid that they are regarded as incompetent English teachers by their students, teachers feel apprehensive when the students have trouble doing the tasks, teachers feel anxious when they make spelling errors, and teachers feel apprehensive when they are not acclaimed by the students.

#### Antecedents of Teachers' Apprehension

**Figure 1:**
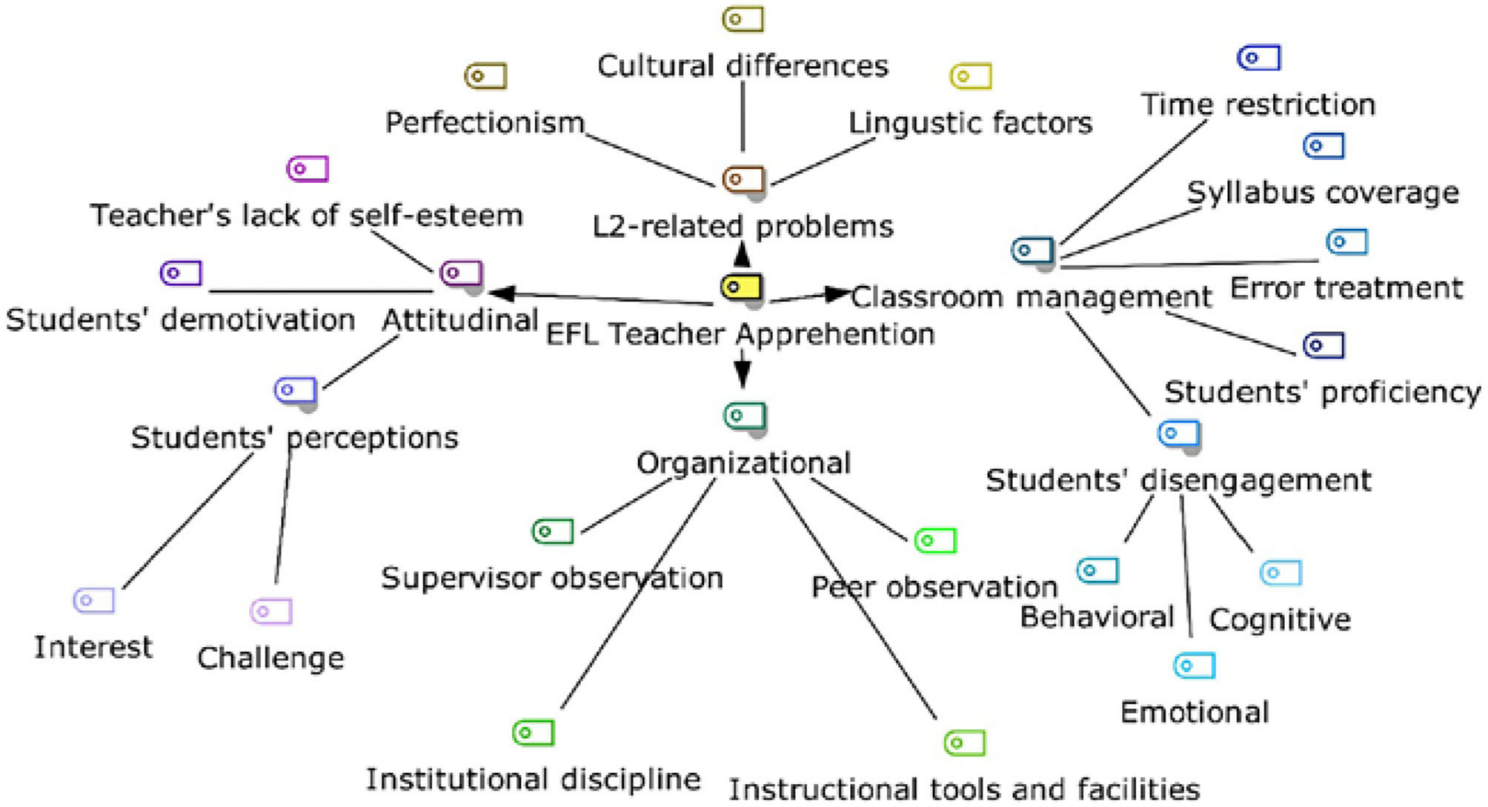
The model of the ramifications of teachers' apprehension (Goldast et al., 2021, p. 7).

Because teachers' well-being seems of outmost importance, a model for the antecedents of teachers' apprehension was raised by Goldast et al. (2021) that indicated that teachers' apprehension can be classified into four classifications that have been shown in Figure 1.

As can be seen in the raised model, antecedents of teachers' apprehension have been classified into four categories: organizational factors including the following examples, when a teacher is supervised by a mentor or supervisor, when a teacher ought to obey institutional discipline, when a teacher has difficulty using the facilities or institutional tools, and when a teacher is observed by his colleagues. The second category is attitudinal factors that can be exemplified in this way: when teachers suffer from low self-esteem, when students do not show much interest in the subject-matter, and when students feel demotivated to be engaged in the class activities. The third factor is L2-related factors including linguistic factors such as the level of difficulty, cultural differences that cause trouble learning a language, and perfectionism which is viewed as negative in this context since one will not start a task if they have had perfect ideas to complete a task comprehensively. Finally, the last category is classroom management including the following examples, when a teacher is pressurized by time limitation that should be allocated in teaching or by the syllabus that should be covered every single session, how students' errors are treated by the teachers, students' level of proficiency is what puts a strain on teachers in that the higher the level, the more apprehensive a teacher may feel to deal with their questions, and students' feeling behavioral, cognitive, emotional disengaged in the activities. This model clearly shows the important role of teachers' apprehension in all aspects of a learning context.

### Classroom Engagement

Engagement is an inseparable part of the learning process and a multifold phenomenon. It has been categorized into different classifications: Behavioral engagement such as the effort; emotional engagement such as high levels of enthusiasm which is linked with low levels of anxiety and boredom; cognitive engagement such as the usage of learning strategy and self-regulation; agentic engagement such as the amount of conscious effort so that the learning experience would be enriched (Veiga et al., 2014; Hiver et al., 2021). Amongst the aforementioned categories, the one which is strongly important in the learning process is behavioral engagement in that it is relevant to the actual recognition of an individual's learning talents (Dörnyei, 2019).

Another possibility that can be viewed is to consider engagement from two other aspects, internal and external. The former implies how much time and effort is allocated to the process of the learning while the latter entails the measures that are taken at the institutional level so that the resources would be dealt with along with other options of learning and services for support which encourage the involvement in activities leading to the possible outcomes such as consistency and satisfaction (Harper and Quaye, 2009). Much attention is deserved to be paid to engagement since it is perceived as a behavioral means with which students' motivation can be realized and as a result, development through the learning process can occur (Jang et al., 2010). Active involvement should be strengthened in L2 classes to prevent disruptive behaviors and diminish the valence of emotions that are negative such as feeling anxious, frustrated, and bored.

It has been claimed by some writers (Skinner, 2016) that disengagement itself does not happen frequently in educational settings due largely to the fact that it is related to extreme behaviors, and it is when another phrase disaffection can be considered significant. Disaffection is characterized by disinterest, aversion, resignation, and reduced effort. Therefore, our perception of boredom as a complex emotion can be enhanced, and it can be dealt with more systematically if boredom is viewed through the following factors, disengagement, and disaffection (Derakhshan et al., 2021). In language learning context and use, engagement with learning a language is a cognitive, affective, and/or social process where the learner is viewed as the agent and language is regarded as the object. The learner is engaged:

- “Cognitively: the engaged individual is alert, complete attention is focused, and their own knowledge is constructed.
- Affectively: the engaged individual has a positive, intentional, willing, and independent approach toward the language and/or what it represents.
- Socially: the engaged individual is interactive and initiating” (Svalberg, 2009, p. 247).

### The Relationship Between Teaches' Apprehension and Classroom Engagement

As it has been believed both teachers' positive attributes and negative attributes can have an effect on students' engagement (Greenier et al., 2021). However, detrimental effects are the only impacts that are left when teachers feel apprehensive and solicitous. Irreparable damage is caused by teachers' apprehension since they cannot provide students with opportunities to express themselves and to reach their pinnacle with regard to the learning process. To be precise, a closer look can be taken through the items of teachers' apprehension scale and the way they impact students' engagement (Ghanizadeh et al., 2020). The first item which falls under the category of L2-related factors is: I am anxious when I have to deal with unfamiliar idioms or expressions in English; when the teacher himself could not leave his comfort zone and struggle to learn new words, how students are supposed not to be terrified by making mistakes and learning materials through trial and error. Item 26 of the STAS is: I feel anxious when words escape me. Forgetting and making mistakes are inseparable parts of learning a new language and they inevitably happen when one is making endeavors to learn a new language. When teachers feel so, students cannot have the bravery to show themselves and continue talking when they forget a word and they feel embarrassed, so as a consequence, they are less likely to be engaged in the activity. The third item which falls under the category of attitudinal factors is: I am not confident in speaking English. To speak a language, confidence needs to be built and it takes high self-esteem when one intends to start speaking a new language. Therefore, when teachers themselves are not confident enough to speak a new language, how their students can be encouraged to overcome language barriers and start speaking a new language without feeling shy and it undoubtedly causes a decrease in the amount they are willing to engage in the classroom activities. Item 25 which falls under the category of organizational factors in this scale is: When my students do not actively participate in class activities, I feel apprehensive. Students' not participating in the activities shows that there might be something wrong with teachers' teaching methods, teachers' stroke, teachers' credibility, teachers' care, teachers' immediacy or students' mental or physical health (Xie and Derakhshan, 2021). What aggravates the situation in this context is that the more apprehensive a teacher feel, the less engaged students are to take part in class activities. The last item of STAS, number 33 is: I feel anxious when I am not praised by the students. It has been told that a teacher should be intrinsically motivated so as not to need someone' s acclaim. When the teachers are required to be praised by their students to feel better about their jobs, students are not expected to rely on such teachers and be willing to engage in the class activities. The conclusion that can be drawn out of the above-mentioned points is that teachers' apprehension and students' classroom engagement are negatively correlated.

## Implications and Further Suggestions for Research

This study aimed to highlight the role of teachers' apprehension in students' engagement since it is the students' engagement that results in successful academic achievements. Teachers' negative feelings such as stress, anxiety, and apprehension are said to affect students' engagement negatively as they are psychological mind-body problems in that one's body is highly affected by some mental issues like depression and apprehension. Teachers' apprehension causes one to feel weak, humiliated, and threatened. It is the reason why this issue is of great value. The more stressed, anxious, and apprehensive a teacher feels, the less engaged the students are in class. In prior studies what has been stressed is the role of students' feelings on students' engagement throughout the class, despite the fact that teachers may have a more crucial impact on students' involvement, particularly those who do not have an outgoing personality. To fill this gap, this study has been done to delve into more details about the relationship between these two variables. It goes without saying that there are some limitations considering this subject. First and foremost, there should be a study to recognize the differentiation between teachers' apprehension and teachers' anxiety even though they might seem the same in the educational context. Secondly, voracious researchers should put effort into practice to address the issue of teachers' apprehension and some solutions should be put forward to resolve it and provide teachers with better well-being since it is not enough to raise a topic and consider it from different aspects. What seems necessary is that teachers' apprehension should be mitigated to see students be more engaged in the classroom activities. Last but not least, the relevance between teachers' apprehension and introverted or extroverted students' engagement can be studied to discover which groups will be more affected by teachers' feeling apprehensive and it will assuredly pave the way for students to know themselves better when it comes to the educational area and they may be motivated to rebuild a new personality by which both their learning process and their personal life is influenced.

## Author Contributions

XL confirms being the soul author of the manuscript and approved for this draft for submission to Frontiers.

## Funding

This study was sponsored by 2017 Project of Young Excellent & Talent Scholars of University of Science and Technology Beijing. It is part of the outcomes of the project Research on flipped classroom of college English education based on pan learning strategy (Project No. 06200041), which is sponsored by Central higher education fundamental research funds. It is also part of the outcomes of USTB Massive Online Open Course Project (Project No. KC2021ZXKF21).

## Conflict of Interest

The author declares that the research was conducted in the absence of any commercial or financial relationships that could be construed as a potential conflict of interest.

## Publisher's Note

All claims expressed in this article are solely those of the authors and do not necessarily represent those of their affiliated organizations, or those of the publisher, the editors and the reviewers. Any product that may be evaluated in this article, or claim that may be made by its manufacturer, is not guaranteed or endorsed by the publisher.
